# Systematic Profiling of Essential Fungal Transcription Factors Uncovers Ezt1 as a Central Pathobiological and Morphogenic Regulator in *Cryptococcus neoformans*

**DOI:** 10.21203/rs.3.rs-9581746/v1

**Published:** 2026-06-02

**Authors:** Yong-Sun Bahn, Seung-Heon Lee, Sheng Sun, Yu-Byeong Jang, Seong-Ryong Yu, Jin-Tae Choi, Yeseul Choi, Eui-Seong Kim, Jong-Seung Lee, Joseph Heitman, Kyung-Tae Lee

**Affiliations:** Yonsei University; Yonsei University; Yonsei University; Yonsei University; Yonsei University; Duke University Medical Center; Jeonbuk National University; AmtixBio Co. Ltd; Duke University School of Medicine; Jeonbuk National University

## Abstract

*Cryptococcus neoformans* is a global fungal pathogen that causes fatal cryptococcosis, underscoring the urgent need for novel therapeutics given the limitations of current antifungals. Here, we systematically investigate essential transcription factors (TFs) in *C. neoformans*, focusing on their roles in growth and their potential as drug targets. We developed experimental pipelines to assess growth requirement, essentiality, and function using conditional gene expression, constitutive overexpression, and meiotic spore analysis. Through these systematic analyses, we identify one quasi-essential (growth-required but non-essential) TF, Fhl1, and 13 essential TFs, including three (Ezt1, Ezt2 and Cbf1) that are highly divergent from counterparts in other eukaryotes. Notably, Ezt1 emerged as a central TF regulating over 1,200 genes, controlling growth, antifungal drug and stress responses, sexual development, and virulence. Collectively, our findings define the essential TF landscape of *C. neoformans* and provide a framework for leveraging these regulators, particularly Ezt1, to develop targeted therapies for cryptococcosis.

## Introduction

*Cryptococcus neoformans*, a basidiomycetous fungus within the pathogenic *Cryptococcus* species complex, is responsible for fatal cryptococcosis in humans^1^. Found in diverse natural environments such as soils and avian excreta, *C. neoformans* primarily affects immunocompromised individuals^2^. Infection commences with the inhalation of spores, which can subsequently disseminate to various organs. Remarkably, the fungus can evade or proliferate within alveolar macrophages^3^ and exhibit neurotropism, allowing it to breach the blood-brain barrier (BBB) and cause lethal meningoencephalitis^4,5^. *C. neoformans* accounts for 19% of AIDS-related deaths, with an estimated annual death toll of 112,000^6^.

Cryptococcosis treatment is hindered by the limited availability of antifungal drugs, further complicated by the limited permeability of the BBB. Furthermore, the prolonged use of antifungal medications, coupled with undesirable side effects and limited efficacy, has led to the emergence of drug-resistant strains, underscoring the urgent need for novel anticryptococcal therapies with unique mechanisms of action. Although targeting essential fungal proteins has proven effective in antifungal drug development, it presents challenges due to potential toxicity risks: many of these proteins are conserved between fungi and humans. For example, the essential TOR kinase Tor1 can be inhibited by rapamycin, leading to significant antifungal activity^7–9^. However, its associated cellular toxicity in humans^10,11^ prevents the use of its inhibitors as a viable antifungal strategy. Thus, developing safer antifungal drugs may require targeting evolutionarily less conserved, species-specific essential proteins.

Essential transcription factors (TFs) present promising drug targets due to their evolutionary divergence from other essential signalling and metabolic proteins^12–14^. However, developing TF-targeting drugs poses challenges, as they must disrupt protein-DNA or protein-protein interactions. Despite these difficulties, there have been successful efforts in cancer treatment and other fields^15–17^. Several essential TFs have been identified in fungi, such as heat shock factor 1 (Hsf1), which regulates thermotolerance and heat shock protein expression in various fungal species, including *C. neoformans*, *Saccharomyces cerevisiae*, and *Candida albicans*^18,19^. In humans, HSF1 functions as a master regulator of heat shock protein expression under thermal stress and plays a role in various aspects of cancer^20^. Other essential TFs have been identified in *S. cerevisiae* and *C. albicans*^21–23^.

Our previous systematic analysis of 178 TFs in *C. neoformans* identified 23 likely essential TFs, as these genes could not be deleted despite repeated attempts^12^. Among these, Hsf1 was confirmed to be essential by our prior study^24^. In contrast, five TFs, such as Mig1, Cys3, Cir1, Ecm2201, and Ccd6, were previously identified as quasi-essential or non-essential and partially characterised^25–29^. Therefore, this study systematically examined the remaining 17 putative essential TFs using conditional gene expression, constitutive overexpression, and spore analysis of heterozygous mutants in engineered diploid strains. Our approach confirmed the essentiality of 13 TFs and elucidated their pathobiological roles both in vitro and in vivo. Notably, Ezt1 emerged as a key transcriptional regulator controlling more than 1,200 genes, many of which are involved in the pathobiology and reproduction of *C. neoformans*. This research functionally characterised an unprecedented number of essential TFs in the global fungal meningitis pathogen, paving the way for potential novel anti-cryptococcal therapies.

## Results

### Strategy for the identification and functional analysis of essential transcription factors

In our study, we focused on 17 previously uncharacterised essential TF candidates (Supplementary Table 1). For TFs already annotated in FungiDB or assigned by homology to *S. cerevisiae*, names and rationales are listed in Supplementary Table 1. For the TFs we newly named, we used domain-based or functional classification: we named *HLH7* (helix-loop-helix type 7) and *FZC52* (fungal Zn_2_-Cys_6_ type 52) based on their TF domain family classification, as no clear yeast orthologues could be identified. Lastly, *EAT1* (essential APSES TF 1), *EZT1* and *EZT2* (essential zinc-finger TFs 1 and 2) were named due to their classification as essential TFs, as elucidated later in this paper.

**Figure 1:**
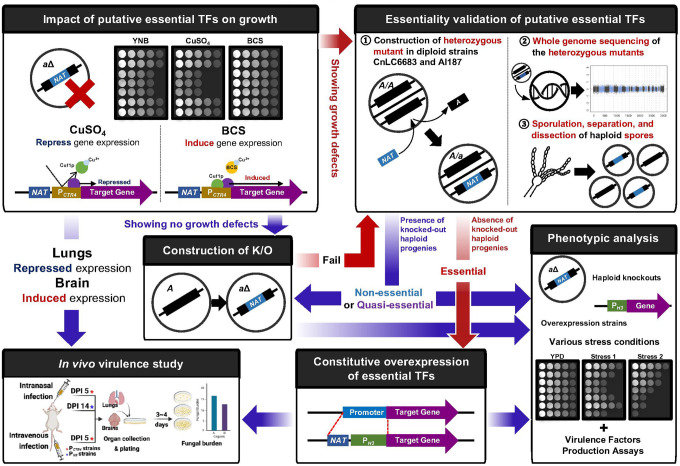
Schematic representation of the strategy used for assessing cryptococcal essential transcription factors (TFs). Essential TFs were identified using both *CTR4* promoter replacement and meiotic spore analysis of heterozygous mutants. Their functional roles were further examined through in vitro phenotypic analysis of constitutive overexpression strains and deletion mutants, as well as in vivo virulence assays using *CTR4* promoter-driven conditional expression and *H3* promoter-driven constitutive overexpression strains in a murine systemic cryptococcosis model. This images for in vivo virulence study and whole genome sequencing were created with BioRender.

We employed two complementary strategies to assess the essentiality of the TFs (Fig. 1). First, we generated conditional gene expression strains for each TF by replacing their native promoters with the copper-repressible *CTR4* (copper transporter gene 4) promoter^30^. TFs exhibiting impaired growth in repressive conditions were classified as ‘growth-required’, while those without growth defects were subjected to further gene deletion attempts. While the *CTR4* promoter replacement approach only indicates whether a target gene is required for growth, it does not confirm the essentiality of the TFs. To resolve this, we constructed heterozygous mutants for spore analysis of all essential TF candidates, following a reported method based on engineered diploid platform strains CnLC6683 (KN99 *MAT*α/*MAT***a**)^31^ and AI187 (*ADE2*/*ade2 URA5*/*ura5 MAT*α/*MAT***a**)^32^. The next steps involved whole-genome sequencing (WGS) to avoid misinterpretations arising from unintended genetic anomalies, such as aneuploidy, chromosomal rearrangements and segmental duplication/deletions.

For WGS-validated heterozygous mutants, we then used two complementary spore isolation methods—spore dissection and spore enrichment—together with genotypic analysis to identify haploid progeny. Target genes were classified as essential TFs if no viable spores carried the disrupted allele alone (Fig. 1). To minimise the effect of background mutations, we primarily analysed heterozygous mutants in CnLC6683, a marker-free strain derived from fusion of KN99α and KN99**a**^31^, by spore dissection directly from basidiospore chains, followed by genotyping. To analyse larger numbers of spores than would be feasible by dissection, and TFs for which genetically validated CnLC6683-based heterozygous mutants could not be obtained, we additionally performed spore enrichment method for AI187-derived heterozygous mutants using Percoll^®^ density-gradient centrifugation^33^. Haploids were isolated using auxotrophic markers and then genotyped by PCR using primers internal to the deleted gene, as described in Materials and Methods. Together, these two approaches enabled a more precise and comprehensive assessment of TF essentiality.

As it is impossible to elucidate the function of essential genes through gene deletion analysis, we developed constitutive overexpression strains utilising the histone *H3* promoter to analyse the function of these putative essential TFs. Subsequently, we assessed their phenotypic traits under 28 distinct in vitro growth conditions. For in vivo phenotypic analysis, we employed a fungal burden assay in a murine model of systemic cryptococcosis, utilising wild-type, constitutive overexpression, and *CTR4* promoter replacement strains (Fig. 1), as previously documented^34^.

### Uncovering essential transcription factors in C. neoformans

**Figure 2:**
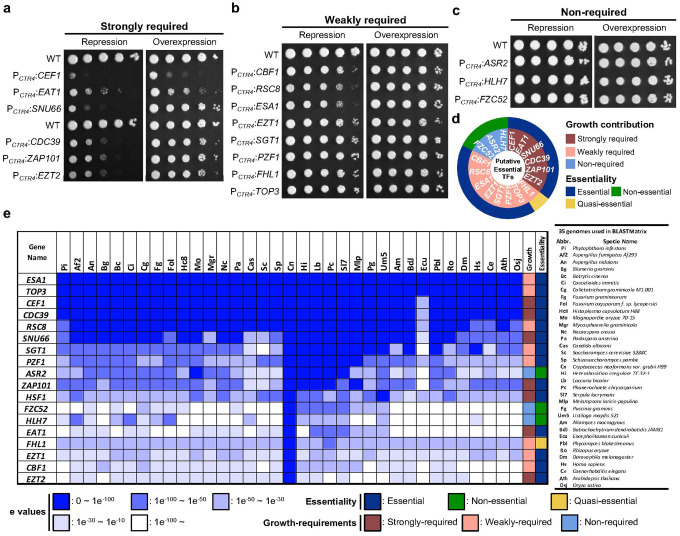
Essentiality assessment of 17 putative essential TFs in *C. neoformans*. (A-C) The wild-type (WT; H99) and *CTR4* promoter replacement strains were cultured overnight at 30°C in liquid YPD medium, serially diluted (1 to 10^4^), and spotted onto YPD plates containing 25 mM CuSO_4_ for repression or 200 mM BCS for induction of the putative essential TF. The plates were incubated at 30°C for 3 days and photographed. *CTR4* promoter replacement strains of *CEF1*, *EAT1*, *SNU66*, *CDC39*, *ZAP101*, and *EZT2* exhibited notable growth defects when CuSO_4_ was added, which were partially rescued when BCS was added to the medium, indicating that these TFs are strongly required for the growth of *C. neoformans*. (B) Using the same experimental method as in (A), eight TFs (*CBF1*, *RSC8*, *ESA1*, *EZT1*, *SGT1*, *PZF1*, *FHL1*, and *TOP3*) were classified as weakly required, and three TFs (*ASR2*, *HLH7*, and *FZC52*) (C) were determined to be non-required for the growth of *C. neoformans*. (D) Spore analysis of heterozygous mutants confirmed that 13 TFs are essential for *C. neoformans* viability, with *FHL1* classified as quasi-essential as it is required for fungal growth. (E) A BLAST matrix of the putative essential TFs was generated using the Comparative Fungal Genomics Platform (http://cfgp.riceblast.snu.ac.kr). Abbreviations (Abbr.) are indicated next to the matrix.

Among the 17 putative essential TFs, analysis of the constructed *CTR4* promoter replacement strains (Supplementary Fig. 1) showed that six TFs (*CEF1*, *EAT1*, *SNU66*, *CDC39*, *ZAP101* and *EZT2*) are strongly required for the growth, whereas eight TFs (*CBF1*, *RSC8*, *ESA1*, *EZT1*, *SGT1*, *PZF1*, *FHL1* and *TOP3*) are weakly required for growth (Fig. 2a and 2b). The remaining three TFs (*ASR2*, *HLH7*, and *FZC52*) exhibited no apparent growth defect in the presence of CuSO_4_ (Fig. 2c and 2d).

We next generated heterozygous mutants in engineered diploid platform strains (CnLC6683 and AI187) for all 17 TFs for subsequent spore analysis (Supplementary Figs. 2, 3, and 4). For most targets, we obtained at least one heterozygous isolate from each diploid background whose WGS profile confirmed loss of a single allele, with no additional truncations or copy-number alterations in the genomic region surrounding the target gene. However, for two TFs (*CBF1* and *TOP3*), WGS-validated intact isolates were obtained only in the AI187 background, whereas for two others (*CEF1* and *FZC52*) such isolates were obtained only in the CnLC6683 background. From 56 heterozygous mutants, we isolated and analysed 1,543 spores: 720 spores from CnLC6683-based spore dissection and 823 spores from AI187-based spore dissection or enrichment (examples on Supplementary Fig. 5). These analyses indicated that 13 TFs are essential for viability, with the exception of *HLH7*, *ASR2*, *FHL1* and *FZC52*. Haploid *NAT*-positive progeny were recovered from self-filamentations of *HLH7/hlh7*, *ASR2*/*asr2*, *FHL1*/*fhl1*, and *FZC52*/*fzc52* mutants, confirming that these genes are not essential. Although a small number of *NAT*-positive progeny were obtained for the 13 essential TFs, all appeared to be aneuploid, as retention of the wild-type allele was verified by internal PCR. Collectively, these results demonstrate that 13 of the 17 putative essential TFs are essential in *C. neoformans*, whereas *FHL1* is required for growth but dispensable for viability, consistent with classification as a quasi-essential gene (Fig. 2d and Supplementary Table 1).

BLAST (Basic Local Alignment Search Tool) analyses indicated that except for the seven proteins (Cbf1, Zap101, Eat1, Ezt1, Ezt2, Hlh7, and Fzc52), all TFs are essential in at least one model fungus among *S. cerevisiae*, *Candida albicans*, *Nakaseomyces glabratus* and *Schizosaccharomyces pombe*^35–40^ (Supplementary Table 1). Notably, the six TFs with the highest sequence homology (Esa1, Top3, Cef1, Cdc39, Rsc8, and Snu66) were essential and comparatively conserved across diverse eukaryotes (Fig. 2e). According to their functional annotations, these evolutionarily conserved essential TFs are primarily components of fundamental cellular machinery. Specifically, Cef1 (a pre-mRNA splicing factor) and Snu66 (a U4/U6.U5 tri-snRNP-associated protein 1) are critical components of the spliceosome complex, rendering them indispensable for proper RNA processing^41^. Additionally, Cdc39 (a CCR4-NOT transcription complex subunit), Rsc8 (an RSC chromatin remodelling complex subunit), and Esa1 (a histone acetyltransferase) serve as key regulators of transcriptional control and chromatin remodelling^42–44^. Furthermore, Top3 functions as a DNA topoisomerase III, essential for maintaining DNA topology^36^. By contrast, the three most divergent TFs—Cbf1, Ezt1 and Ezt2—remained essential despite substantial sequence divergence (Fig. 2e).

### In vitro and in vivo phenotypic profiling of essential cryptococcal TFs

**Figure 3:**
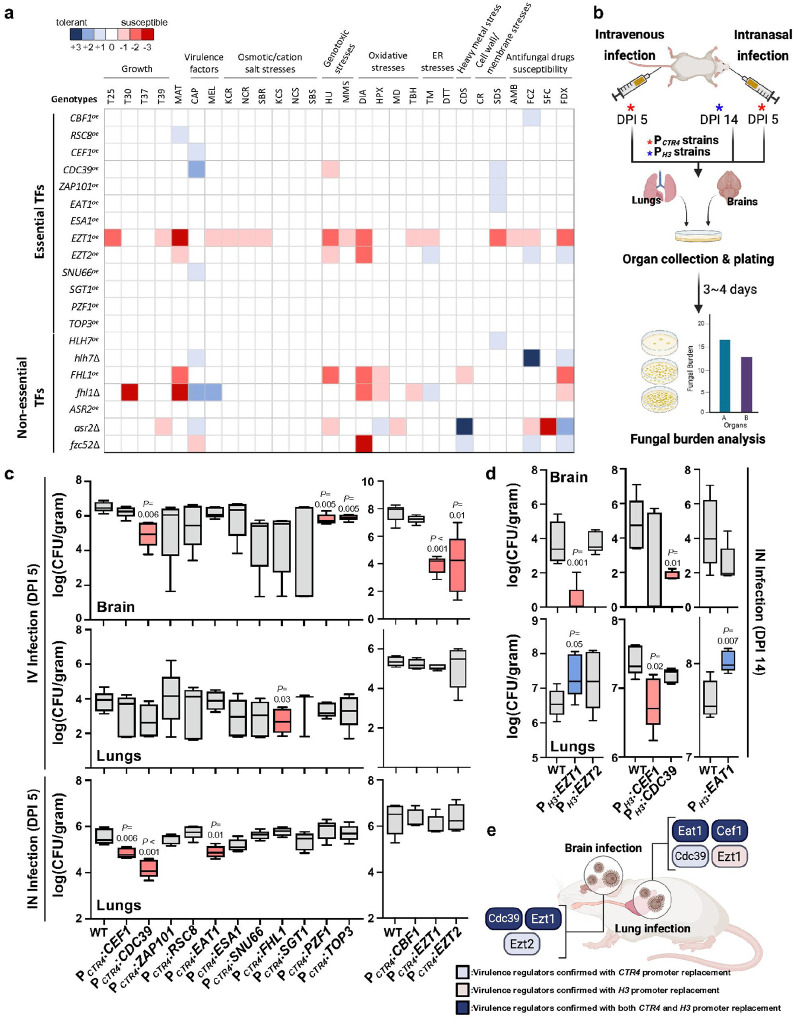
Phenotypic and pathogenicity profiling of essential TFs in *C. neoformans*. (A) In vitro phenotypic traits of essential TF overexpression strains and non-essential TF knockout/overexpression strains were assessed under various growth environments, stress conditions, antifungal treatments, and virulence-factor-inducing conditions. In the heat map, red and blue indicate phenotypic reduction and enhancement, respectively, with intensity (strong, intermediate, and weak) represented by color gradients, as defined in the legend above. Abbreviations: T25 (25°C), T30 (30°C), T37 (37°C), T39 (39°C), MAT (mating), CAP (capsule production), MEL (melanin production), KCR (YPD + 1.5 M KCl), NCR (YPD + 1.5 M NaCl), SBR (YPD + 2 M sorbitol), KCS (YP + 1 M KCl), NCS (YP + 1 M NaCl), SBS (YP + 2 M sorbitol), HU (hydroxyurea), MMS (methyl methanesulphonate), DIA (diamide), HPX (hydrogen peroxide), MD (menadione), TBH (*tert*-butyl hydroperoxide), TM (tunicamycin), DTT (dithiothreitol), CDS (cadmium sulphate), CR (Congo red), SDS (sodium dodecyl sulphate), AMB (amphotericin B), FCZ (fluconazole), 5FC (5-flucytosine), FDX (fludioxonil). (B) Schematic representation of fungal burden assays in murine models of systemic cryptococcosis. *CTR4* promoter replacement strains were used to assess fungal burden following intravenous (IV) and intranasal (IN) infections. The *H3* promoter overexpression strains were tested under IN infection to evaluate the impact of transcriptional regulation on fungal burden. This image was created with BioRender. (C) Fungal burden analysis of *CTR4* promoter replacement strains in murine lungs and brains after IV and IN infections. Box plots indicate fungal CFU levels for each strain. TFs influencing brain and lung burdens are highlighted in red. Statistical significance of difference was determined by two-tailed Welch’s *t*-tests using Prism 11.0 and *P* values were indicated above or below the box plot. (D) Fungal burden in lungs and brain of overexpression strains for *EZT1*, *EZT2*, *RSC8*, *CEF1*, *CDC39*, and *EAT1* following IN infection. (E) Overview diagram illustrating the essential TFs regulating brain and lung infections in *C. neoformans*. Essential TFs such as *EZT1* and *CDC39* were associated with both brain and lung infection, while *EAT1* and *CEF1* played roles in lung infection. This image was created with BioRender.

To functionally characterise the essential TFs, we constructed constitutive overexpression strains driven by the histone 3 (H3) promoter (Supplementary Fig. 6). For the non-essential TFs, phenotypes were assessed using haploid knockout progeny obtained by spore dissection from *HLH7/hlh7*, *ASR2/asr2* and *FHL1/fhl1* heterozygous mutants in the CnLC6683 background, together with an H99-background knockout strains for *FZC52* (Supplementary Fig. 7). Using these overexpression strains alongside the corresponding knockout strains, we performed a comprehensive in vitro phenotypic analysis to elucidate TF function in *C. neoformans* (Fig. 3a).

We tested 28 conditions spanning temperature sensitivity, mating efficiency, virulence factor production (capsule and melanin), stress responses and antifungal drug susceptibility (Fig. 3a and Supplementary Fig. 8). While most overexpression strains showed minor or no phenotypic changes, *EZT1oe* and *EZT2oe* strains exhibited more pronounced phenotypes (Fig. 3a). Notably, *EZT1* overexpression markedly impaired growth at 25°C and 39°C and increased susceptibility to osmotic, genotoxic and oxidative stresses (diamide and organic peroxide), tunicamycin, SDS and antifungal agents (amphotericin B, fluconazole and fludioxonil). By contrast, *EZT2* overexpression increased susceptibility to hydroxyurea and diamide, while enhancing resistance to tunicamycin and antifungal agents (fluconazole and fludioxonil).

*FHL1*, a quasi-essential gene, displayed dynamic phenotypic effects in both the overexpression and knockout contexts. The *FHL1oe* strain showed increased susceptibility to hydroxyurea and heavy metal CdSO_4_. In contrast, the *fhl1*Δ mutant exhibited enhanced melanin and capsule production, increased susceptibility to organic peroxide, and heightened resistance to tunicamycin (Fig. 3a). Notably, both *FHL1oe* and *fhl1*Δ strains displayed impaired growth under oxidative stress (diamide and H_2_O_2_) and reduced mating efficiency. These results underscore the importance of tightly balanced *FHL1* expression for adaptation to diverse stresses and for sexual development.

To elucidate the roles of quasi-essential and essential TFs in the pathogenicity of *C. neoformans*, we performed a fungal burden assay in a murine model of systemic cryptococcosis, using *CTR4* promoter replacement strains (Fig. 3b). This approach was based on previous work showing that *CTR4* promoter-driven transcription is repressed during lung infection but induced during brain infection^34^. Five days after intravenous (IV) infection, fungal burdens in the brain and lungs were quantified. In the brain, several *CTR4* promoter replacement strains (*CDC39*, *PZF1*, *TOP3*, *EZT1* and *EZT2*) showed significant reductions in fungal burden compared with the wild-type, with *CDC39*, *EZT1* and *EZT2* displaying the most pronounced effects (Fig. 3c). In the lungs following IV infection, only the *FHL1 CTR4* promoter-replacement strain showed a significant reduction relative to the wild type (Fig. 3c). In addition, intranasal (IN) infection revealed that three targets (*CEF1*, *CDC39*, and *EAT1*) showed reduced lung fungal burdens measured at day 5 compared to the wild-type strain (Fig. 3c), indicating that these TFs influence the early stage of pulmonary infection.

We next reassessed virulence using *H3* promoter-driven constitutive overexpression strains (Fig. 3b), focusing on the essential TFs *CEF1*, *CDC39*, *EAT1*, *EZT1* and *EZT2*. The *CEF1oe* strain exhibited a reduced lung fungal burden compared with the wild-type strain, whereas the *EAT1oe* strain showed an increased lung fungal burden (Fig. 3d). In addition, *CDC39* overexpression significantly reduced brain fungal burden, and *EZT1* overexpression decreased fungal survival in the brain but increased lung colonisation (Fig. 3d). These findings indicate that a subset of essential TFs exerts organ-specific control of cryptococcal virulence. Specifically, Eat1 acts as a positive regulator of pulmonary infection, whereas the balanced regulation of *CEF1* expression is required for effective lung infection. Moreover, the reduced brain survival observed for *EZT1oe* and *CDC39oe* strains suggests that Ezt1 and Cdc39 contribute to the regulation of brain infection.

Collectively, our in vitro and in vivo phenotypic analyses suggest that the essential TFs Cdc39 and Ezt1 are key regulators of both brain and lung infection, whereas Cef1 and Eat1 primarily modulate pulmonary infection in *C. neoformans*. (Fig. 3e).

### Transcriptional networks governed by essential TFs Ezt1, Cbf1, and Ezt2 in C. neoformans

**Figure 4:**
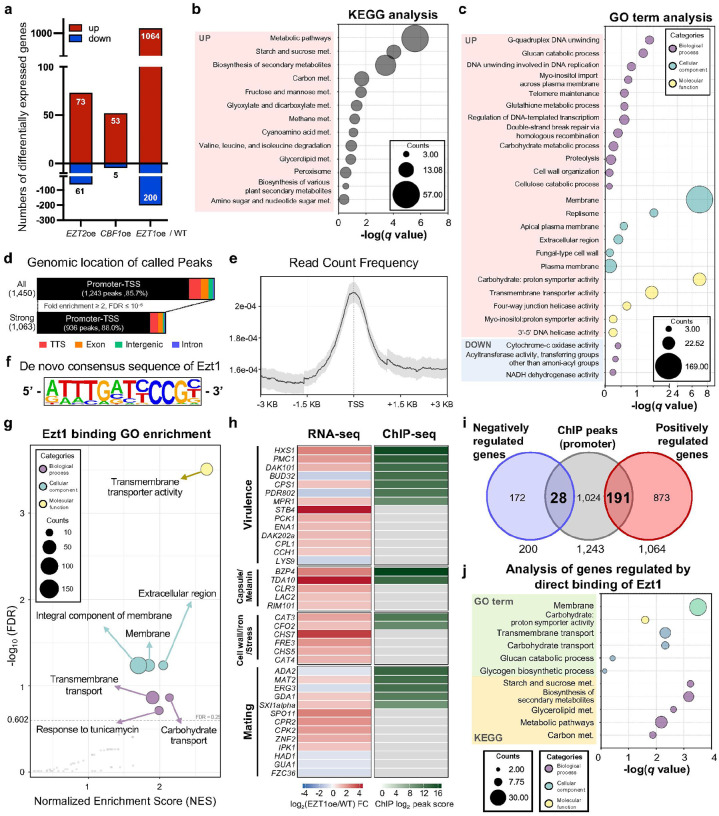
Transcriptional network regulated by *EZT1*, *EZT2*, and *CBF1* overexpression. (A) RNA sequencing (RNA-seq) analysis showing the number of differentially expressed genes (DEGs) in *EZT1oe*, *CBF1oe*, and *EZT2oe* strains compared to the wild-type strain (WT). (B) Kyoto Encyclopedia of Genes and Genomes (KEGG) pathway analysis showing the functional categories of genes regulated by *EZT1*. (C) Gene ontology (GO) analysis revealing that *EZT1* overexpression broadly upregulates genes involved in biological processes, cellular components, and molecular functions. (D) Genomic distribution of Ezt1-binding peaks annotated by HOMER. Of the 1,450 reproducible peaks identified, 1,243 (~85.7%) were located in promoter–transcription start site (TSS) regions; 936 high-confidence promoter peaks (fold enrichment > 2, q < 10^−5^) were used for downstream motif analysis. (E) Metagene plot showing that Ezt1 occupancy is sharply centred on the TSS. The shaded grey region indicates the 95% confidence interval estimated by bootstrap resampling. This plot was generated using ChIPseeker in R. (F) De novo consensus binding motif identified at Ezt1-bound sites by HOMER. (G) Volcano plot of pre-ranked gene set enrichment analysis (FGSEA) of GO terms across genes ranked by Ezt1 promoter-proximal binding intensity. Each point represents a GO term; point size indicates gene set size, and fill color indicates ontology category. The dashed horizontal line denotes FDR = 0.25. This plot was generated using fgsea in R. (H) Heatmap showing Ezt1 ChIP-seq peak scores (right, green) and RNA-seq log2 fold changes in *EZT1oe* versus WT (left, red–blue) for manuscript-referenced virulence-, capsule/melanin-, cell wall/iron/stress-, and mating-associated genes. Grey tiles indicate the absence of a promoter-proximal Ezt1 peak. Within each category, genes with Ezt1 binding are ordered by peak score (top), followed by genes without binding ordered by log2 fold change. This heatmap was generated using R. (I) Integration of RNA-seq and ChIP-seq data identified 219 genes directly regulated by Ezt1, including 191 induced and 28 repressed genes. (J) GO analysis and KEGG pathway analysis of the genes regulated by direct binding of Ezt1.

We next sought to delineate the transcriptional networks governed by evolutionarily divergent essential TFs Cbf1, Ezt1 and Ezt2 (Fig. 2e), two of which (Ezt1 and Ezt2) are linked to virulence. We performed RNA sequencing (RNA-seq) on *EZT1oe*, *CBF1oe*, and *EZT2oe* strains and compared their transcriptomes with that of the wild-type strain under basal growth conditions. Notably, *EZT1* overexpression elicited extensive transcriptional remodelling, altering the expression of 1,264 genes—substantially more than observed upon overexpression of *EZT2* or *CBF1* (Fig. 4a, Supplementary Data 2 and Supplementary Fig. 9a). Principal component analysis (PCA) and the interquartile range (IQR) plot further showed that *EZT1* overexpression induced broader and higher-magnitude transcriptomic changes than overexpression of either *CBF1* or *EZT2* (Supplementary Fig. 9b and c), underscoring the wide regulatory scope of Ezt1 in *C. neoformans*.

KEGG pathway analysis of the differentially expressed genes (DEGs) indicated that Ezt1 regulates diverse metabolic programmes, including starch and sucrose metabolism, biosynthesis of secondary metabolites, carbon metabolism, amino acid degradation, glycerolipid metabolism, peroxisome and amino sugar/nucleotide sugar metabolism (Fig. 4b). In contrast, Cbf1 and Ezt2 affected a more restricted set of pathways: with Cbf1 impacting metabolic pathways, secondary metabolite biosynthesis, and amino acid degradation, while Ezt2 preferentially targeted pathways such as tyrosine metabolism (Supplementary Fig. 9d). Gene ontology (GO) term analysis using DAVID further showed that Ezt1 broadly upregulates genes involved in multiple biological processes, cellular components, and molecular functions (Fig. 4c). While Cbf1 and Ezt2 similarly regulated specific processes, their overall regulatory scope was markedly narrower than that of Ezt1 (Supplementary Fig. 9d).

Notably, Ezt1 regulated a wide range of virulence-associated genes in *C. neoformans*. Among the upregulated genes, fourteen (*STB4, HXS1*, *PMC1*, *ZNF2*, *PCK1*, *ENA1*, *DAK101*, *DAK202a*, *CPL1*, *CPS1*, *IPK1*, *RIM101*, *MPR1* and *CCH1*) have previously been implicated in virulence^13,45–58^, along with genes linked to capsule biosynthesis (*TDA10*, *BZP4*, *CLR3* and *RIM101*)^12,59–61^. *EZT1* overexpression also induced coordinated transcriptional changes in other virulence-related modules, including melanin biosynthesis and regulation (*LAC2* and *BZP4*)^62,63^, cell wall remodelling (*CHS7* and *CHS5*)^64^, iron acquisition (*FRE3*, *CFO2* and *STR1*)^65,66^, and oxidative stress defence (*CAT3* and *CAT4*)^67^. Furthermore, *EZT1* overexpression altered the expression of several sexual-development-associated genes (*SXI1*α, *CPR2*, *ZNF2*, *SPO11* and *CPK2*)^68–72^. Conversely, *EZT1* overexpression downregulated several pathogenicity-associated genes, including *PDR802*, *LYS9*, and *BUD32*^73–75^, as well as mitochondrial-encoded electron transport genes such as cytochrome c oxidase III and NADH dehydrogenase subunits (Supplementary Data 2). Collectively, these findings highlight Ezt1 as a central, lineage-specific TF in *C. neoformans* that coordinates key virulence factors and related biological functions.

### Transcriptional network directly regulated by the essential TF Ezt1 in C. neoformans

To further investigate Ezt1 as a transcriptional regulator, we examined its subcellular localisation by tagging the C-terminus of Ezt1 with the mRuby3 fluorescent protein (Supplementary Fig. 10a). The Ezt1-mRuby3 strain phenocopied the wild-type strain under all conditions tested, indicating that C-terminal tagging with mRuby3 did not impair Ezt1 function (Supplementary Fig. 10b). Fluorescence microscopy showed that Ezt1-mRuby3 was predominantly nuclear during mid-lag and early-to-mid exponential growth, but relocalised to the cytoplasm from late exponential phase through stationary phase (Supplementary Fig. 10c). These observations suggest a role for Ezt1 in regulating growth stage-dependent transcriptional programmes.

To validate Ezt1 as a nuclear DNA-binding protein, we generated an Ezt1-4×FLAG strain by tagging the C-terminus of Ezt1 with 4×FLAG epitopes (Supplementary Fig. 10a). This strain phenocopied the wild-type strain, confirming that the Ezt1-4×FLAG protein is functional (Supplementary Fig. 10b and 10d). Using this strain, we performed chromatin immunoprecipitation sequencing (ChIP-seq) and identified 1,450 reproducible binding peaks (FDR < 0.05) by three-way intersection of three biological replicates. Of these, 1,243 (85.7%) were annotated to promoter-transcription start site (TSS) regions by HOMER, corresponding to 1,243 target genes, and 936 peaks with fold enrichment > 2 and FDR < 10^− 5^ were defined as high-confidence promoter binding sites (Fig. 4d and Supplementary Data 3). Metagene analysis showed that Ezt1 occupancy was sharply centred on the TSS (Fig. 4e). De novo motif analysis of these peaks revealed a conserved 12-bp motif (5’–ATTTGATYCCGY–3’, Y = C/T; *P* = 1 × 10^−111^; Fig. 4f and Supplementary Data 3). Collectively, these results indicate that Ezt1 directly regulates a substantial fraction of the cryptococcal transcriptome.

Pre-ranked gene set enrichment analysis (FGSEA), which assesses enrichment across a ranked gene list, revealed that strong Ezt1 occupancy was preferentially directed toward genes encoding transmembrane transporter activity, extracellular region components, and integral membrane components, together with transmembrane transport and carbohydrate transport processes (Fig. 4g). To examine how Ezt1 binding intersects with the virulence-, capsule/melanin-, cell wall/iron/stress-, and mating-associated genes identified in our RNA-seq analysis above, we constructed a gene-level integration map combining RNA-seq log_2_Fold Changes with Ezt1 ChIP-seq peak scores (Fig. 4h; Supplementary Data 2 and 3). Prominent Ezt1 occupancy was observed at promoters of key virulence factors (*HXS1*, *PMC1*, *DAK101*, *BUD32*, *CPS1*, *PDR802*, and *MPR1*)^13,45,49,50,53,58,74^, capsule/melanin regulators (*BZP4* and *TDA10*)^13,60^, and a subset of mating-associated genes (*ADA2*, *MAT2*, *ERG3*, and *SXI1α*)^68,71,76,77^. Although *ADA2* did not meet the predefined RNA-seq threshold, it showed a modest but statistically significant decrease in expression (log_2_FC = −0.55). Other mating-associated genes with altered expression in *EZT1oe* (*SPO11*, *CPK2*, *ZNF2*, and *HAD1*)^14,69–71^ did not show detectable Ezt1 binding within their promoters, suggesting that their transcriptional changes may be mediated indirectly, downstream of Ezt1.

Building on this gene-by-gene view, we next integrated the RNA-seq and ChIP-seq datasets to identify the full complement of genes directly regulated by Ezt1 at a genome-wide scale. This analysis identified 219 genes directly regulated by Ezt1, including 191 induced and 28 repressed genes (Fig. 4i and Supplementary Data 3). Functional enrichment of the direct targets recapitulated the membrane-transport signature observed in the binding-intensity analysis (Fig. 4g): induced targets were enriched for membrane components and carbohydrate-related transport and metabolism (Fig. 4j). Collectively, our transcriptomic and ChIP-seq analyses establish Ezt1 as an essential transcriptional regulator that coordinates metabolic and virulence-associated programmes, thereby contributing to both pathogenicity and cellular homeostasis in *C. neoformans*.

### Ezt1 as a repressor of sexual reproduction and morphogenic transitions in the pathogenic Cryptococcus species

**Figure 5:**
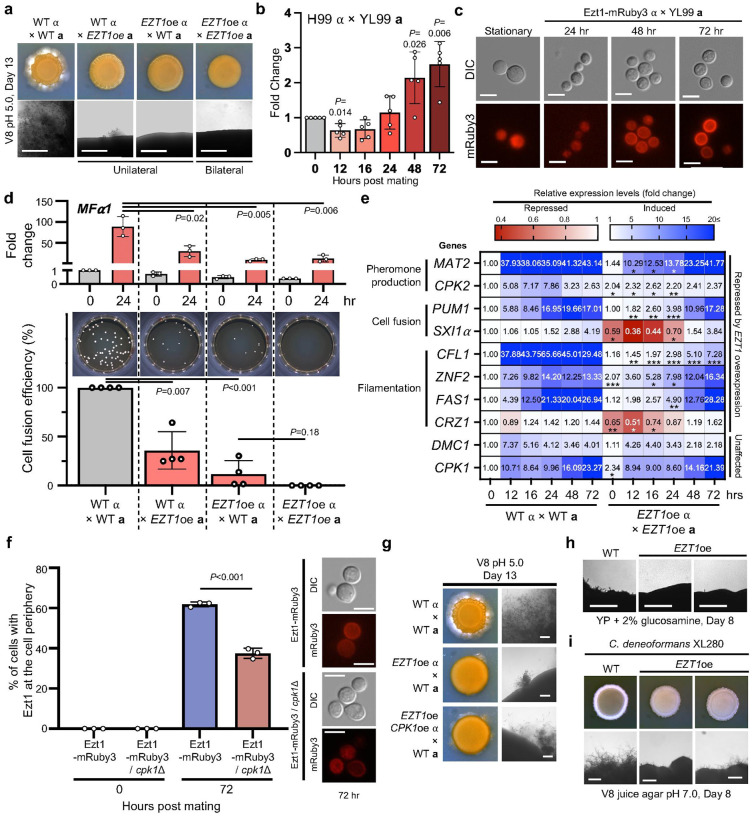
Ezt1 functions as a repressor of sexual reproduction and morphogenic transitions in *C. neoformans*. (A) Overexpression of *EZT1* in both *MAT*α and *MAT***a** strains significantly inhibited mating under unilateral and bilateral conditions (scale bar: 500 μm). (B) qRT-PCR analysis showing dynamic *EZT1* expression during mating. *EZT1* expression decreased up to 16 h post-mating but increased after 48 h. Data are represented as mean ± SD (standard deviation). Statistical significance was analysed at each time point relative to the starting point using a two-tailed one-sample *t*-test compared to 0 hr time point (=1) with Prism 11.0 and *P* values were stated above (*n*=5). (C) Fluorescence microscopy of the Ezt1-mRuby3 strain showing Ezt1 translocation to the cell periphery after mating (scale bar: 5 μm). (D) *EZT1* overexpression repressed the expression of the mating pheromone gene *MFα1*, reducing cell fusion efficiency. Data are represented as mean ± SD. Statistical significance for mating pheromone production was analysed using an two-tailed unpaired *t*-test with WT bilateral mating condition (*n*=3), and that for cell fusion assay was analysed using a two-tailed one-sample *t*-test to compare with wild-type unilateral settings and with two-tailed unpaired *t*-test for *EZT1oe* α× WT **a** and *EZT1oe* bilateral with Prism 11.0 (*P* values were stated together; *n*=4). (E) *EZT1* overexpression delayed or reduced the expression of key mating-related genes, while genes such as *DMC1* and *CPK1* remained unaffected. In bilateral mating conditions, the temporal expression levels of each gene were statistically analysed by comparing them to the corresponding time points in wild-type mating using two-tailed unpaired *t*-test, and two-tailed one-sample *t*-test for 0-hour time point with Prism 11.0 (*n*=5; * *P* < 0.05; ** *P* < 0.01; *** *P* < 0.001, *P* values of each gene and time points were listed at Supplementary data 6). (F) Deletion of *CPK1* in the Ezt1-mRuby3 strain inhibited Ezt1 translocation to the membrane during mating, indicating that Cpk1 modulates Ezt1 localisation. Data are represented as mean ± SD, and statistical significance was determined using two-tailed Welch’s *t*-test with Prism 11.0 with *P* values stated together (*n*=3). (G) Co-overexpression of *CPK1* in the *EZT1oe* strain partially rescued the severe mating defect caused by *EZT1* overexpression, suggesting that Cpk1 regulates Ezt1 function. (H) *EZT1* overexpression reduced self-filamentation on glucosamine media (scale bar = 200 μm). (I) In *C. deneoformans* XL280, *EZT1* overexpression abolished unisexual reproduction (scale bar = 200 μm).

ChIP-seq and transcriptome analyses together suggest that Ezt1 is linked to the regulation of mating-associated genes, supporting a role for this factor in sexual differentiation in this pathogenic fungus. Consistent with this, overexpression of *EZT1* drastically reduced mating even in unilateral settings (Fig. 3a). To further support the transcriptional evidence linking Ezt1 to mating-associated pathways, we additionally constructed an *EZT1* overexpression strain in the *MAT***a** YL99 strain background^78^, driven by the *H3* promoter (Supplementary Fig. 11a). The *MAT***a** P_*H3*_:*EZT1* (*EZT1oe* **a**) strain phenocopied the *MAT*α P_*H3*_:*EZT1* (*EZT1oe* α) strain, indicating that Ezt1 performs conserved functions in both mating types (Supplementary Fig. 11b). *EZT1* overexpression nearly abolished mating in both unilateral and bilateral mating set-ups (Fig. 5a), consistent with Ezt1 functioning as a mating repressor. We quantified *EZT1* expression levels at multiple time points during mating by qRT-PCR. *EZT1* expression was significantly reduced up to 16 h post-mating but subsequently increased after 48 h (Fig. 5b). Notably, at 48 h and 72 h post-mating, Ezt1 translocated to the cell periphery, consistent with a potential shift in its regulatory activity during mating progression (Fig. 5c).

Initiation of sexual reproduction typically involves induction of mating pheromones followed by cell fusion. We found that *EZT1* overexpression significantly repressed *MFα1* expression, correlating with reduced cell-fusion efficiency (Fig. 5d). Moreover, in bilateral mating (*EZT1oe* α × *EZT1oe* **a**), *EZT1* overexpression significantly reduced expression of *SXI1*α and *CRZ1*, both of which are required for hyphal formation^68,79^. Induction of other key mating-associated genes involved in pheromone production, cell fusion and filamentation—including *CFL1*, *MAT2*, *ZNF2, CPK2*, *PUM1* and *FAS1*^71,80^—was delayed or reduced during the early phase (~ 24 h) of sexual reproduction, whereas *DMC1* and *CPK1* expression was unchanged (Fig. 5e). Collectively, these data indicate that Ezt1 acts as a transcriptional repressor during the early stages of sexual differentiation in *C. neoformans*.

We next investigated the upstream regulator controlling Ezt1 during mating. Given the overlap in function, we hypothesised that Cpk1, a mitogen-activated protein kinase (MAPK) that governs pheromone production and cell fusion, regulates Ezt1. To test this, we deleted *CPK1* in the *EZT1-mRuby3* strain (Supplementary Fig. 12) and monitored Ezt1 localisation during mating. Notably, deletion of *CPK1* inhibited the Ezt1 localisation to the cell periphery observed at 72 h post-mating, indicating that Cpk1 promotes Ezt1 translocation (Fig. 5f). A previous study in the closely related species *Cryptococcus deneoformans* reported that *CPK1* disruption did not alter *EZT1* transcript levels during sexual reproduction^81^, suggesting that Cpk1 does not regulate Ezt1 at the transcriptional level. Given that Ezt1 acts as a repressor during the early stage of mating, we therefore postulated that Cpk1 modulates Ezt1 post-translationally rather than by altering its expression. To further test this model, we generated a double overexpression strain by introducing a *CPK1* overexpression construct into the *EZT1oe* strain (Supplementary Fig. 12b). Under mating conditions, the severe mating defect caused by *EZT1* overexpression was partially rescued by *CPK1* overexpression, supporting the conclusion that Cpk1 modulates Ezt1 function during mating (Fig. 5g).

In addition to its role in bisexual reproduction, we assessed the impact of Ezt1 on self-filamentation and unisexual differentiation. *EZT1* overexpression reduced self-filamentation on glucosamine media, which induces this developmental programme^79^ (Fig. 5h). Moreover, in *C. deneoformans* strain XL280, which undergoes robust unisexual differentiation, *EZT1* overexpression abolished this process (Fig. 5i and Supplementary Fig. 13). Collectively, these findings indicate that Ezt1 functions as a central negative regulator of sexual reproduction and morphogenic transitions across the pathogenic *Cryptococcus* species complex.

### Conserved essential roles of Ezt1 in the Cryptococcus species complex

**Figure 6:**
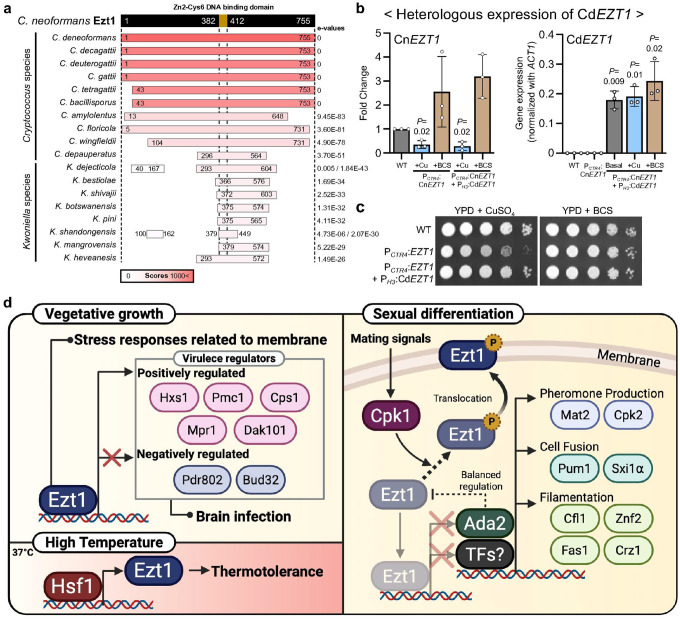
Conserved essential roles of Ezt1 in the *Cryptococcus* species complex. (A) BLASTP analysis showing structural conservation of Ezt1 across *Cryptococcus* species. In *C. depauperatus*, only the Zn2-Cys6 DNA-binding domain in the middle region is conserved, with a significantly shorter alignment length compared to other species. (B) Functional complementation assay. Overexpression of *C. depauperatus EZT1* (*CdEZT1*) was tested in the *C. neoformans* conditional *EZT1* expression strain (P_*CTR4*_:*EZT1*) under copper-repressed conditions. qRT-PCR confirmed *CdEZT1* expression and conditional regulation of Cn*EZT1*. Data are represented as mean ± SD. Statistical significances of *CnEZT1* repression and *CdEZT1* overexpression were determined by comparison with wild-type expression levels using two-tailed one-sample *t*-test with Prism 11.0 compared to WT (=1), and *P* values for were stated above (*n*=3). (C) Growth assay showing that *CdEZT1* overexpression successfully restores wild-type growth of the P_*CTR4*_:*EZT1* strain under Cn*EZT1* repressed conditions, indicating functional conservation of Ezt1 across the *Cryptococcus* species complex. (D) Proposed model of Ezt1 regulation in growth, stress responses, thermotolerance, virulence, and morphogenic development. Under normal growth conditions, Ezt1 controls membrane stress responses, positively regulating virulence factors (Hxs1, Pmc1, Cps1, Mpr1, and Dak101) while negatively regulating others (Pdr802 and Bud32), contributing to brain infection. At high temperature (37°C), Hsf1 activates Ezt1 to promote thermotolerance. During mating, Ezt1-Cpk1 interactions contribute to Ezt1 translocation, ensuring balanced regulation of pheromone production (Mat2 and Cpk2), cell fusion (Pum1 and Sxi1α), and filamentation (Cfl1, Znf2, Fas1, and Crz1). The interaction between Ezt1, Ada2, and other transcription factors remains to be fully elucidated. This image was created with BioRender.

Because Ezt1 is among the most sequence-divergent essential cryptococcal TFs, we assessed its conservation across fungi using BLAST. Ezt1 appears conserved only within *Cryptococcus* species. Notably, Ezt1 is highly conserved among the pathogenic *Cryptococcus* species complex—including *C. neoformans*, *C. deneoformans*, *C. decagattii*, *C. deuterogattii*, *C. gattii*, *C. tetragattii* and *C. bacillisporus*—with e-values close to zero. Ezt1 orthologues were also detected in non-pathogenic *Cryptococcus* species, with significant sequence similarity (Fig. 6a). Notably, in *Cryptococcus depauperatus*, conservation was largely restricted to the central region containing the Zn2-Cys6 DNA-binding domain, resulting in markedly shorter alignment than in other *Cryptococcus* species (Fig. 6a). In *Kwoniella* species, which are closely related to *Cryptococcus*, overall similarity to *C. neoformans* Ezt1 was substantially reduced, particularly outside the DNA-binding domain (Fig. 6a).

We next tested whether the truncated Ezt1 orthologue from *C. depauperatus* (CdEzt1) could functionally complement *C. neoformans* Ezt1 (CnEzt1). To this end, we overexpressed *CdEZT1* in a *CTR4*-regulated conditional *EZT1* expression strain (P_*CTR4*_:*EZT1*) by integrating a P_*H3*_:*CdEZT1* allele into a previously characterised safe-haven locus^82^ (Supplementary Fig. 14). We confirmed the successful regulation of *CnEZT1* under *CTR4* conditions and constitutive *CdEZT1* overexpression (Fig. 6b). Strikingly, *CdEZT1* overexpression restored wild-type growth of the P_*CTR4*_:*EZT1* strain under *EZT1*-repressing conditions (Fig. 6c), supporting the conclusion that Ezt1 fulfills a conserved essential function within the *Cryptococcus* species complex.

## Discussion

In this study, we identified and functionally characterised 13 essential TFs (Esa1, Top3, Cef1, Cdc39, Rsc8, Snu66, Sgt1, Pzf1, Zap101, Eat1, Cbf1, Ezt1 and Ezt2) in *C. neoformans*, a leading cause of fungal meningitis worldwide. These factors were uncovered by prioritising 17 putative essential TFs that had not been disrupted in previous genome-wide functional analyses of cryptococcal TFs^12^. Our findings are notable because, prior to this study, Hsf1 was the only essential TF to have been functionally characterised in *C. neoformans*^24^. We also established an experimental pipeline to define essentiality and interrogate in vitro and in vivo functions of essential cryptococcal proteins. To determine and validate essentiality, we used two complementary approaches: a conditional gene expression system and spore analysis of heterozygous mutants generated in engineered diploid strains. To investigate function, we constructed constitutive overexpression strains and performed a murine fungal burden assay using both conditional expression and overexpression strains. We propose that this integrated strategy provides a robust platform for the identification and functional characterisation of essential cryptococcal proteins.

Conditional repression from the *CTR4* promoter indicated that fourteen TFs are required for growth. Subsequent spore analyses showed that thirteen of these are essential for viability in *C. neoformans*, whereas loss of Fhl1 resulted in impaired but viable growth, consistent with a quasi-essential role. By contrast, the remaining three TFs showed no growth defects upon *CTR4* promoter-mediated repression, and spore analyses confirmed that they are non-essential. Together, these results indicate that growth phenotypes obtained with the *CTR4* promoter-based conditional expression system correlate closely with gene essentiality in *C. neoformans*. Despite this internal consistency, comparison with a previous transposon sequencing (Tn-seq) study revealed several discrepancies in essentiality assignments^83^. That study identified *ZAP101*, *EZT1* and *TOP3* as non-essential genes^83^. Moreover, *FHL1* was reported as essential in the Tn-seq study, yet our findings indicate that it is quasi-essential. These differences highlight how experimental conditions and genetic approaches can influence essentiality assignments. For example, genes required for spore germination may be scored as essential in our platforms even if they are dispensable at other life-cycle stages. Further work will be required to determine whether these genes are universally required throughout the cryptococcal life cycle, underscoring the value of multifaceted approaches for accurate essentiality assessment.

Our in vivo virulence assays, using murine infection models, with both *CTR4* promoter-based conditional expression and H3 promoter-driven constitutive overexpression strains, identified several essential TFs associated with *C. neoformans* pathogenicity: *EZT1*, *EZT2* and *CDC39* for brain infection, and *EZT1*, *EAT1*, *CEF1* and *CDC39* for lung infection. The inhibitory effects of *EZT1* and *CDC39* overexpression on brain infection were consistently observed across both approaches. Moreover, conditional repression of *CDC39* reduced fungal burdens in the lungs, whereas *EZT1* overexpression increased lung colonisation, indicating that these essential TFs exert multifaceted roles during the infection cycle. Similarly, the positive role of *EAT1* in pulmonary colonisation was supported by both approaches. For *CEF1*, both repression and overexpression reduced lung fungal burden, indicating that tightly balanced *CEF1* expression is required for efficient pulmonary colonisation; this is consistent with our finding that both *CEF1* repression and overexpression markedly impaired in vitro growth. Further studies will be required to define the molecular mechanisms by which these TFs regulate virulence. Our results also showed that *FHL1* repression reduced lung colonisation, suggesting that quasi-essential TFs may still exert substantial tissue-specific effects. Extending classical virulence assays to the newly identified quasi- or non-essential TFs Asr2, Fhl1, Fzc52, and Hlh7 should therefore provide additional insights into how these dispensable regulators contribute to cryptococcal pathogenicity. Collectively, delineating the roles of both essential and non-essential TFs may inform the development of targeted antifungal strategies that exploit vulnerabilities specific to brain or lung infection.

A key finding of this study is the identification of Ezt1, an essential TF specific to *Cryptococcus* species. Although recent Tn-seq data suggested that Ezt1 may be non-essential in *C. neoformans*, the same dataset notably showed the absence of transposon insertions within the central DNA-binding domain and the nuclear localisation signal of Ezt1^83^. Together with our results, this pattern supports the conclusion that Ezt1 is an essential TF in this pathogenic fungus. Ezt1 is a fungal-specific Zn2-Cys6 TF conserved largely within the *Cryptococcus* species complex, yet absent or highly divergent in closely related taxa. Although evolutionarily divergent proteins are often assumed to be dispensable, our data demonstrate that Ezt1 is essential and regulates a broad range of pathobiological processes in *C. neoformans*. *EZT1* overexpression altered the expression of 1,264 genes, ~ 60% of which were previously unannotated in *C. neoformans* and other fungi (Supplementary Data 2), underscoring the unique regulatory scope of Ezt1. Consistent with this, ChIP-seq identified Ezt1 binding at 1,243 genes, reinforcing its role as a broad-spectrum regulator of the *C. neoformans* transcriptome. Notably, *EZT1* overexpression markedly reduced brain infection by *C. neoformans*. This effect is likely attributable to the significant downregulation of *PDR802*, a TF required for crossing the BBB^74^, and *BUD32*, a KEOPS complex component implicated in tRNA modification and pathogenicity^73^ (summarised in Fig. 6d).

Our integrated ChIP-seq and RNA-seq analyses revealed that Ezt1 directly regulates genes involved in diverse metabolic pathways and in membrane structure and function. Consistent with this, in vitro phenotypic profiling indicated that Ezt1 is required for tolerance to temperature extremes, osmotic stress and membrane stress—phenotypes closely linked to membrane fluidity, integrity and composition^84–86^. Notably, *EZT1* overexpression increased sensitivity at 25°C and 39°C, underscoring a key role for Ezt1 in thermal adaptation. Interestingly, recent ChIP-seq data showed that Hsf1, another essential TF, directly binds to the *EZT1* promoter under heat shock conditions (37°C and 40°C)^87^ (our manuscript submitted elsewhere), suggesting that Hsf1 may act upstream of *EZT1* to promote membrane stability and cell survival at elevated temperatures (Fig. 6d). Additionally, the antifungal agents to which *EZT1* overexpression increased susceptibility (amphotericin B, fluconazole and fludioxonil) are known to target processes associated with membrane stability^88–90^. Together, these observations support the conclusion that Ezt1 is critical for maintaining membrane function and promoting stress adaptation in *C. neoformans*.

Our findings indicate that Ezt1 functions as a central morphogenetic regulator in *C. neoformans*. Consistent with this, transcriptomic and ChIP-seq analyses showed that Ezt1 directly represses genes involved in sexual differentiation, and qRT-PCR analysis of gene expression changes during mating confirmed its regulatory role at the early stages of sexual differentiation. We further found that the Cpk1 MAPK acts upstream of Ezt1, promoting its localisation to the cell periphery and modulating its function during mating (Fig. 6d). Because Cpk1 and Ezt1 do not appear to regulate one another’s transcript levels during mating^81^, Cpk1-mediated regulation of Ezt1 is likely post-transcriptional or post-translational, for example, through phosphorylation. This model is consistent with the proposal by Jang et al. that an uncharacterised TF downstream of Cpk1 regulates Mat2, a key transcriptional regulator of mating-associated genes^71^. As Ezt1 does not directly bind these mating genes in our ChIP-seq dataset, it is plausible that other Ezt1-dependent TFs could contribute to their regulation. Indeed, Ezt1 bound to the promoter of the transcription factor *ADA2*, whose deletion impairs mating^12^, and modestly repressed its expression under basal conditions. Notably, previous histone H3 ChIP-seq data indicate that Ada2-dependent chromatin regulation at the *EZT1* promoter represses its expression^76^, suggesting a reciprocal regulatory interplay between Ada2 and Ezt1 that may help ensure appropriate mating progression. Collectively, these results support a model in which Ezt1 acts as a global morphogenetic regulator in *C. neoformans* (Fig. 6d). Taken together, these findings raise the possibility that Ezt1 integrates metabolic, virulence-associated, and morphogenetic programmes in *C. neoformans*, which may partly underlie its essential role in this pathogen.

The mechanisms by which Ezt1, a *Cryptococcus*-specific protein, evolved into an essential TF within this species complex remain unclear. BLAST analyses indicate that the Zn2-Cys6 DNA-binding domain of Ezt1 is highly conserved among *Cryptococcus* species. Our functional data suggest that Ezt1 orthologues fulfil conserved essential roles across the *Cryptococcus* species complex. Notably, whereas Zn2-Cys6 motifs are typically located near the N-terminus of fungal TFs^91^, this domain resides in the central region of Ezt1. By contrast, the N- and C-terminal regions of Ezt1 are highly divergent, which may contribute to *Cryptococcus*-specific transcriptional networks. ChIP-seq revealed widespread Ezt1 occupancy across the *C. neoformans* genome, consistent with a role in regulating a broad set of targets. This supports a model in which Ezt1 acts as a general transcriptional regulator that coordinates essential metabolic pathways and core biological processes in *Cryptococcus*. Further studies defining structure-function relationships and mapping interactions between Ezt1 and other components of the transcriptional machinery will be important for elucidating its regulatory mechanisms and evaluating its potential as a therapeutic target.

Despite the evolutionary divergence of TFs, which makes them attractive antifungal drug targets, they have often been overlooked due to the inherent challenges associated with inhibiting their activity. Nonetheless, TF-targeting strategies have been pursued, particularly in cancer and other diseases^15–17^. In *C. albicans*, for example, the TF Upc2, which regulates sterol synthesis and is responsive to azole treatment, has been exploited to screen for small molecules that inhibit its expression or DNA binding^92^. In this context, we propose that Ezt1, Eat1 and Ezt2 merit further investigation as potential targets for anti-cryptococcal drug development, given that these TFs are structurally divergent, essential and crucial for *C. neoformans* virulence. Furthermore, it is also important to consider the therapeutic potential of the highly conserved essential TFs identified in this study, including Esa1, Top3, Cef1, Cdc39, Rsc8, and Snu66, as they govern fundamental cellular machinery required for cell growth and survival. Although targeting conserved proteins often raises concerns regarding host toxicity, exploiting subtle structural divergences or fungus-specific interactions with their binding partners could facilitate the development of selective inhibitors that minimise host toxicity. Alternatively, targeting downstream genes regulated by these essential TFs—particularly those critical for virulence—may represent a complementary and tractable strategy. To validate the therapeutic potential and virulence impact of these essential TFs *in vivo*, future studies should employ tightly regulatable conditional expression systems, such as the tetracycline-repressible knockdown system^93^. This approach would overcome the potential expression fluctuations of the *CTR4* promoter in the host and provide precise temporal control of gene expression during cryptococcal infection. In summary, our work provides a foundation for developing new therapeutic approaches against the globally important pathogen *C. neoformans* by leveraging essential fungal TFs.

## Materials and Methods

### Ethics statement

Animal experiments were performed in accordance with the ethical guidelines of the Institutional Animal Care and Use Committee of Jeonbuk National University (NON2023–132). The committee approved all vertebrate studies, and all experiments adhered to the experimental ethics guidelines.

### Construction of C. neoformans CTR4 promoter replacement strains

*CTR4* promoter replacement cassettes were generated by double-joint PCR (DJ-PCR), as previously described^94^, using nourseothricin-resistant marker (nourseothricin N-acetyltransferase; *NAT*). The primer pair B354/B355 was used to amplify the *NAT*-*CTR4* promoter with pNAT-*CTR4* as a template. Approximately 800 bp of the 3′ region of the promoters and 800 bp of the 5′ region of the exons of the target genes were amplified using the primer pairs *CTR4*_L1/*CTR4*_L2 and *CTR4*_R1/*CTR4*_R2, respectively, with H99 genomic DNA (gDNA) as the template (Supplementary Data 4). After amplification of the 3′ promoter regions, 5′ exon regions, and *NAT*-*CTR4* in the first round of PCR, primer pairs *CTR4*_L1/SM2 and *CTR4*_R2/SM1 were used to amplify 5′ and 3′ regions of the *NAT*-*CTR4*-split cassettes in the second round of PCR. All primers and generated *CTR4* promoter replacement strains were listed in Supplementary Data 4 and 5. The targeted insertion cassettes were then introduced into the *C. neoformans* H99 using biolistic transformation, as described previously^95^. The H99 strain was cultured in liquid yeast extract-peptone-dextrose (YPD) medium (1% yeast extract, 2% peptone, and 2% dextrose) for 16 h at 30°C in a shaking incubator, spun down, resuspended in 5 ml distilled water, plated onto YPD agar plates containing 1 M sorbitol, and further incubated at 30°C for 3 h. Purified targeted insertion cassettes were prepared with 600 μg of 0.6 μm gold microcarrier beads (Bio-Rad, CA, USA) using calcium chloride and spermidine and delivered into H99 using a particle delivery system (PDS-100, Bio-Rad). Plates were incubated at 30°C for 4 h to restore the disrupted cell membrane, scraped, and spread on YPD agar plates containing 100 μg/ml nourseothricin. Stable transformants were selected and further screened for correct insertion by diagnostic PCR. Finally, Southern blot analysis was performed to validate the correct genotypes of the promoter-replacement strains using gene-specific probes.

### Construction of C. neoformans heterozygous mutants

Single copies of the TFs were deleted in the diploid *C. neoformans* AI187 and CnLC6683 through homologous recombination using gene disruption cassettes containing signature-tagged *NAT*. The gene disruption cassettes were generated by DJ-PCR with the primer pairs listed in the Supplementary Data 4. PCR amplified the 5′- and 3′-flanking regions of the target TFs with the primer pairs L1/L2 and R1/R2, using gDNA as a template, while the M13Fe/M13Re pair was used to amplify signature-tagged *NAT*. Split-gene deletion cassettes were then generated using the primer pairs L1/SM2 and R2/SM1 and introduced to the parental diploids via biolistic transformation, as described above. Stable transformants were selected, screened with diagnostic PCR, and confirmed through Southern Blot analysis with gene-specific probes. All of the generated heterozygous mutants were listed in Supplementary Data 5.

### Construction of C. neoformans constitutive overexpression strains

The essential TFs were subjected to overexpression by replacing their native promoters with the histone *H3* promoter using targeted insertion, similar to the *CTR4* promoter replacement method. The primer pairs *CTR4*_L1/*CTR4*_L2 and *H3*_R1/*CTR4*_R2 were used to amplify the 3′ region of the promoter and the 5′ region of the exons, respectively, with H99 gDNA as a template (primers listed in Supplementary Data 4). The primer pair B4017/B4018 was used to amplify the *NAT*-*H3* or *NEO* (G418/neomycin resistance marker)-*H3* promoter, using pNAT-H3 or pNEO-H3 as the template. The second round of PCR was conducted using primer pairs *CTR4*_L1/SM2 and *CTR4*_R2/SM1 to construct the *NAT*-*H3*-split cassettes. These generated cassettes were introduced into the H99 strain via biolistic transformation. Stable transformants were selected on YPD plates containing nourseothricin, screened with diagnostic PCR, and confirmed through Southern blot analysis with gene-specific probes. Two independent overexpression strains had their genotypes and overexpression levels confirmed by Southern blot analysis and quantitative reverse transcription PCR (qRT-PCR), respectively. All the overexpression strains used in this study are listed in Supplementary Data 5.

### Expression analysis by quantitative RT-PCR

To monitor the gene expression levels in conditional expression and constitutive overexpression strains, qRT-PCR was performed. For *CTR4* promoter replacement strains, cells were cultured in liquid YPD medium at 30°C for 16 h in a shaking incubator. The cultures were then diluted to an OD_600_ of 0.2 with fresh YPD, YPD containing 40 μM CuSO_4_, and YPD containing 200 μM of bathocuproinedisulfonic acid (BCS; a copper chelator). The mixtures were cultured at 30°C in a shaking incubator for 8 h, collected by centrifugation, and subjected to overnight lyophilisation. For constitutive overexpression strains, cells were cultured in liquid YPD medium at 30°C for 16 h in a shaking incubator, diluted to an OD_600_ of 0.2 with fresh YPD, and grown further at 30°C in a shaking incubator until reaching an OD_600_ of ~ 0.8. Cell cultures were collected by centrifugation and lyophilised overnight. For mating-associated gene expression, *MAT*α and *MAT***a** strains were cultured in liquid YPD medium at 30°C, washed three times with phosphate-buffered saline (PBS), and combined at equal concentrations (10^8^ cells/mL). A 500 μl aliquot of the cell mixture was plated onto V8 agar medium (5% V8 juice, 4% agar, pH 5.0) and incubated at room temperature in the dark for designated time points. Cells were then scraped and lyophilised overnight. Total RNA was extracted using the easy-Blue RNA extraction kit (iNtRON Biotechnology, Korea), cDNA synthesis was performed using Maxima H Minus RTase (Thermo Scientific, USA) with 5 μg of RNA. qRT-PCR was conducted with gene-specific primers (Supplementary Data 4) on the CFX96 Touch Real-Time PCR Detection System (Bio-Rad), as previously described^71^. Relative gene expression fold change was analysed using the 2^−ΔΔCT^ method^96^, and data were illustrated using Prism 11.0.

### Sporulation, spore separation, and spore analysis of heterozygous mutants

For spore purification and random spore analysis of heterozygous TF mutants constructed in the AI187 background, we followed a previously reported protocol with slight modifications^33,97^. Heterozygous mutants were induced to sporulate on V8 juice agar pH 5.0. Cells were cultured at 30°C for 16 h in a shaking incubator, collected, washed twice with PBS, adjusted to an equal concentration (10^7^ cells/ml), and spotted (3 μl) on V8 juice agar plates. The plates were incubated in the dark for 4 to 6 weeks to induce meiosis and sporulation. Once spores were visible under the microscope, the mixed cell mass, including filaments, basidia, spores, and yeast cells, was suspended in 75% Percoll^®^ (Sigma Aldrich, MO, USA) made isotonic with PBS. The suspensions were then centrifuged at 3,000 rpm for 20 min at 4°C to generate a gradient. Spores formed a band near the bottom of the gradient, while all other cell types remained at the top. The spore band was collected by puncturing the tube wall with a 20 ml tuberculin syringe, followed by centrifugation and two washes with PBS. Spores were diluted 1/100 and spread onto yeast nitrogen base (YNB) medium with amino acids and ammonium sulphate, supplemented with 1 mg/ml uracil, 2 mg/ml adenine, and 1 mg/ml 5-fluoroorotic acid (5-FOA; for excluding diploid *ura5*/*URA5* yeast cells). Plates were incubated at 30°C for 7 days, and the resulting colonies were subjected to mating locus PCR using mating type-specific primer pairs (Supplementary Data 4). Spores with confirmed mating types were used for further analysis. To test genetic markers segregation during meiosis (*ade2*/*ADE2*, *ura5*/*URA5*, *MAT***a**/*MAT*α, and *NAT*), spores were cultured at 30°C in liquid YPD for 16 h and spotted onto the following plates: YPD, YPD + 100 μg/ml nourseothricin, YNB, YNB + 2 mg/ml adenine, and YNB + 1 mg/ml uracil plates. Knockout spores were identified by their growth on YPD + nourseothricin and confirmed by internal PCR using gene-specific primers.

For direct spore dissection analysis, heterozygous TF mutants constructed in the AI187 or CnLC6683 strain backgrounds were sporulated on MS medium (pH 5.8). Spores were mechanically detached through microscopic manipulation using a fibre optic needle spore dissection system, as previously described^97^. After germination, progeny demonstrating growth on YPD + nourseothricin were analysed by internal PCR to confirm gene knockouts as described above.

### Growth and chemical susceptibility test

*C. neoformans* cells were grown at 30°C in liquid YPD for 16 h, serially diluted (1 to 10^4^), and then spotted on YPD plates containing various chemicals to induce stress, as previously reported^12^. The types of stresses tested and chemicals used to induce them were as follows: antifungal drug susceptibility [Fludioxonil (FDX), flucytosine (5-FC), fluconazole (FCZ), and amphotericin B (AMB)], toxic heavy metal stress [cadmium sulphate (CdSO_4_, abbreviated to CDS)], cell wall stress [Congo Red (CR) and sodium dodecyl sulphate (SDS)], oxidative stress [hydrogen peroxide (HPX), tert-butyl hydroperoxide (TBH), menadione (MD), and diamide (DIA)], genotoxic stress [hydroxyurea (HU) and methyl methanesulphonate (MMS)], and ER stress [tunicamycin (TM) and dithiothreitol (DTT)]. Osmotic pressures were induced with 1.5 M NaCl, 1.5 M KCl, and 2 M sorbitol in YPD medium, as well as 1 M NaCl, 1 M KCl, and 2 M sorbitol in YPD without dextrose (YP) medium. The spotted plates were incubated at 30°C and photographed daily for 1 to 5 days using a Bio-Rad Gel-doc imaging system.

### Murine infectivity assays

Animal experiments in this study were conducted at the Core Facility Center for Zoonosis Research (Jeonbuk National University, South Korea). Inbred 6-week-old female BALB/cAnNCrlOri mice, confirmed as SPF/VAF, were purchased from ORIENT BIO INC. (Korea) and acclimated to the breeding environment for one week before the experiment. Infections with *CTR4* promoter-driven conditional expression strains were performed with reference to a previous study^34^. Each strain was inoculated into fresh liquid YPD medium and cultured overnight at 30°C with shaking. An equal number of 5×10^4^ cells in 40 μl of PBS were administered via lateral tail vein injection (IV) or intranasal inhalation (IN). For IN, mice were anaesthetised with isoflurane vapour (Hana Pharm. Co., Ltd., South Korea) at a flow rate of 80 cc/min. Mice were humanely sacrificed 5 days post-infection, and fungal burden was measured in both the brain and lungs (IV model) or in the lungs (IN model).

For the murine infectivity assay using *H3* promoter-driven constitutive overexpression strains, an equal number of 5×10^4^ cells in 40 μl of PBS was administered via IN. Mice were sacrificed 14 days post-infection, and fungal burden was measured in both the brain and lungs. To quantify fungal burden, tissue samples were homogenised in 1 ml of PBS and plated on YPD medium supplemented with 100 μg/ml chloramphenicol. The plates were incubated at 35°C for two days, after which the fungal burden was quantified and expressed as the logarithmic CFU per gram of tissue. Statistical analysis was performed using Welch’s *t*-test on log-transformed fungal burden (CFU/gram) to compare each tested strain with the wild-type control using GraphPad Prism 11.0.

### Transcriptome analysis of the EZT1, EZT2, and CBF1 overexpression strains

The wild-type (H99), P_*H3*_:*CBF1*, P_*H3*_:*EZT1*, and P_*H3*_:*EZT2* strains were grown in YPD liquid medium at 30°C overnight. The cultures were then transferred to fresh YPD liquid medium and incubated until the OD_600_ reached 0.8. Three biologically independent cultured samples were prepared for each strain, and total RNA was purified. A cDNA library was constructed using 1 μg of total RNA for each sample with the Illumina TruSeq RNA library kit v2 (Illumina) and sequenced using the Illumina platform. The reads were processed to remove adaptor and low-quality sequences using Cutadapt v2.4 with Python 3.7.4 with adapter sequence^98^. The reference genome sequence of *C. neoformans* H99 and annotation data were obtained from the NCBI FTP server. The reads were aligned to the genome sequence using Hisat2 v2.2.1 with the Hisat and Bowtie2 algorithm, as previously reported^99^. Hisat2 was performed with the “-p 30” and “-- dta − 1” options, while other parameters were set to default. Aligned reads were converted and sorted using Samtools v0.1.19 with default parameters except for the “-Sb -@ 8” option for converting and the “-@ 20 –m 2000000000” option for sorting^100^. Transcript quantification was performed using the featureCounts command within the Subread package v2.0.8, installed via Bioconda^101^. The parameters used for featureCounts were “-T 4- M—largestOverlap -g gene_id”. Differentially expressed genes (DEG) analysis was performed using DESeq2 v1.24. The volcano plot was illustrated using R v4.1.0, with the cutoff set to more than two-fold changes with *P* < 0.05.

### Chromosomal tagging of Ezt1 with mRuby3 and 4×FLAG

To express Ezt1 fusion proteins tagged with either 4×FLAG or mRuby3 tag, two separate constructs were generated using the following procedure. For the Ezt1-4×FLAG construct, the 3′ exon region was amplified using primer pair B19021/B10922, and the terminator region was amplified with B10923/B10924. For the Ezt1-mRuby3 construct, the 3′ exon region was amplified using B19021/B20540, and the terminator region was amplified with B20541/B19024. The *NAT* marker containing *4×FLAG*-*HOG1t*-*NAT* allele was PCR-amplified with primer pair B5536/B1966, whereas the *mRuby3-HOG1t-NEO* allele was amplified using B16758/M13Re. The amplified 3′ exon, terminator, epitope, and selection marker regions were fused via DJ-PCR; for the 3′ exon, primer pair B10921/B1445 was used for 4×FLAG, and B10921/B1997 for mRuby3. For the terminator, B10924/B1444 were used for 4×FLAG, and B10924/B1886 for mRuby3. Finally, the resulting targeted insertion cassettes were introduced to the H99S strain via biolistic transformation. Correct genotypes were confirmed by performing 3′ junction PCR using primers JOHE12579/B18848, followed by additional validation through Southern blot analysis with an *EZT1*-specific probe amplified using primers B19068/B19069.

### Chromatin immunoprecipitation-sequencing (ChIP-seq) of the Ezt1-4×FLAG strain

The *C. neoformans* Ezt1-4×FLAG strain was cultured in liquid YPD at 30°C overnight. Chromatin extraction was performed as previously reported^59^. The culture was transferred to fresh YPD liquid medium and incubated until the OD_600_ reached 0.8. In vivo crosslinking was achieved by adding 37% formaldehyde to a final concentration of 1%, and the mixture was incubated at 30°C for 5 min with agitation. Crosslinking was quenched by adding 2.5 M glycine (to a final concentration of 300 mM), and the mixture was incubated at 30°C for an additional 5 min with agitation. Cells were collected, washed twice with ice-cold PBS, and once with ice-cold FA lysis buffer (50 mM HEPES pH 7.5, 140 mM NaCl, 1 mM EDTA, 1% Triton X-100, 0.1% sodium deoxycholate, 0.1% SDS, and protease inhibitors), and resuspended in 0.8 ml of ice-cold FA lysis buffer. Cells were homogenised using a bead beater (FastPrep-24 5G, MP Biomedicals) for 5 intervals of 2 min each, with 1 min of cooling on ice between intervals. The lysate was centrifuged for 2 min at 4,000 rpm, and the supernatant was subjected to sonication until the DNA fragment size reached 150–500 bp. A 20 μl aliquot of the chromatin samples was reserved as input control, and DNA was purified using a DNA purification kit (Cosmogenetech, South Korea).

ChIP was performed as previously described^59^. Protein A Dynabeads^®^ (60 μl per sample) were washed twice with ice-cold PBS containing 5 mg/ml bovine serum albumin (BSA). The beads were resuspended in 60 μl of PBS with 5 mg/ml of BSA, and anti-FLAG antibody (Sigma-Aldrich, USA) was added. Each sample was incubated at 4°C overnight with gentle rotation. The antibody-conjugated beads were washed twice with ice-cold PBS containing 5 mg/ml BSA and then aliquoted into four tubes. The extracted chromatin was added to the washed beads, and each sample was incubated at 4°C for 3 h with gentle rotation. After incubation, the beads were washed three times with FA lysis buffer, three times with FA lysis buffer containing 0.5 M NaCl, and once with TE buffer (10 mM Tris-HCl and 10 mM EDTA, pH 8.0), using a magnetic separator (General Electric, USA). DNA was eluted from the beads with 35 μl of elution buffer (50 mM Tris-HCl, pH 8.0, 10 mM EDTA, and 1% SDS) at 65°C for 1 min. The eluted samples were treated with 15 μl of 10% SDS, 1 μl of RNase H, and 3 μl of RNase cocktail (A&T, Ambion, USA) at 65°C overnight. The samples were then treated with 7 μl of proteinase K at 42°C for 90 min to reverse the crosslinking. Reverse-crosslinked DNA was purified using a PCR purification kit (Cosmogenetech, South Korea). Following evaluation of DNA quality by absorbance measurement using a NanoDrop spectrophotometer (Thermo Scientific, USA), sequencing libraries were constructed using the TruSeq ChIP-seq library preparation kit (Illumina, USA). Libraries were sequenced on an Illumina NovaSeq 6000.

### ChIP-seq data processing and binding-site analysis

Raw reads were preprocessed to remove adaptor sequences and filter out low-quality reads using Cutadapt (v2.4; Python 3.7.4). Cleaned reads were aligned to the *C. neoformans* H99 reference genome using HISAT2 (v2.2.1) with default parameters. Aligned reads were converted to BAM format and sorted using SAMtools (v0.1.19). Peak calling from each ChIP-seq replicate was performed using MACS2 (v2.2.7.1), and significant peaks were identified based on a threshold of ≥ 2-fold enrichment and an adjusted *P* value < 0.05. Reproducible Ezt1-binding sites detected in all three biological replicates were extracted using BEDTools (v2.31.1), and the resulting consensus peaks were annotated to the *C. neoformans* H99 genome using HOMER (v5.1). High-confidence promoter-binding sites, used for motif analysis and downstream enrichment tests, were defined as peaks located in promoter–TSS regions with fold enrichment > 2 and q < 10^− 5^. De novo and known motif analyses on the high-confidence peak set were performed using HOMER. Binding profiles were visualised using the Integrative Genomics Viewer (v2.16.1), and metagene analysis around TSSs (± 3 kb) was performed using the ChIPseeker package in R, weighted by peak intensity to emphasise strongly bound sites, with 95% confidence intervals estimated by bootstrap resampling.

To assess the functional categories preferentially associated with strong Ezt1 binding without applying arbitrary peak-score cut-offs, pre-ranked gene set enrichment analysis was performed using the fgsea package in R. HOMER-annotated peaks within ± 2 kb of the nearest TSS were collapsed to the gene level by retaining the maximum peak score per gene, log_2_-transformed, and ranked in decreasing order. GO annotations for C. neoformans H99 were obtained from FungiDB (release 68); some GO terms (e.g., GO:0016021, GO:0055114) have since been deprecated and merged into broader categories in recent GO releases, and their historical names are retained here for clarity. fgsea was run separately for Biological Process, Molecular Function, and Cellular Component ontologies with gene sets filtered to contain 10–500 genes. Multiple-testing correction was performed using the Benjamini–Hochberg procedure applied separately within each ontology, and GO terms with FDR < 0.25 were considered significantly enriched.

### Mating, filamentation, and cell fusion assays

To evaluate bisexual mating efficiency in both unilateral and bilateral settings, wild-type and genetically engineered strains constructed in *MAT*α H99 and *MAT***a** YL99 strain backgrounds were cultured in YPD medium at 30°C for 16 h, washed twice with PBS, and combined at equal concentrations (10^7^ cells/ml). The mixtures were plated onto V8 juice agar (pH 5.0) and incubated in the dark at 25°C for 7 to 14 days. For glucosamine-induced filamentation, H99 and *EZT1oe MAT*α strains were cultured in YPD medium at 30°C for 16 h, washed twice with PBS, and adjusted to a concentration of 10^7^ cells/ml. A 3 μl aliquot of resuspended cells were spotted onto YP + 2% glucosamine plates and incubated at 30°C for 2 days before transferring to 25°C to observe self-filamentation. Filamentous growth was monitored and documented via photography. To induce unisexual reproduction in *C. deneoformans* XL280 and its derived *EZT1oe* strains, cultures were prepared as described above, and 3 μl of cell suspension was plated onto V8 juice agar (pH 7.0). Plates were incubated in the dark at 25°C for 5 to 10 days, and filamentous growth was monitored and photographed using a differential interference contrast microscope ECLIPSE Ni (Nikon, Japan) equipped with DS-QI2 camera and a BX51 microscope (Olympus, Japan) with a SPOT digital camera (Diagnostic Instruments, Inc. USA).

For assessing cell fusion efficiency, we followed an established protocol^102^. Equal volumes of *MAT*α and *MAT***a** control or tested strains, carrying *NAT* or *NEO* markers, respectively, were cultured in liquid YPD medium at 30°C for 16 h and combined at a concentration of 10^7^ cells/ml. A 5 μl aliquot of the mixed cell suspension was plated onto V8 juice agar (pH 5.0) and incubated in the dark at room temperature for 24 h. Cells were collected by scraping and resuspended in 1 ml of distilled water, and a 20 μl aliquot was plated onto a YPD medium supplemented with nourseothricin (100 μg/ml) and G418 (50 μg/ml). Colony counts were recorded after 4 days of incubation in the dark at room temperature.

### Heterologous expression of CdEZT1 in C. neoformans

To construct a *C. neoformans* P_*CTR4*_:*EZT1* strain heterologously overexpressing *Cryptococcus depauperatus EZT1* (*CdEZT1*), the overexpression cassette was integrated into a safe haven region as previously described^82^. The intergenic region between CNAG_00777 and CNAG_00778 was divided into two fragments and amplified by PCR using primer pairs B21128/B21129 and B21130/B21131, respectively. Overlap PCR was then performed to introduce AscI, BaeI, and PacI restriction enzyme sites into the middle of the intergenic region. The JEC21 *H3* promoter (P_*H3*_), amplified with primer pair B21132/B21133, was fused to this intergenic region using overlap PCR. The fused product was assembled into the pHYG vector^103^ using Gibson assembly (New England Biolabs, USA), creating the pHYG-Safe Haven (SH)-P_*H3*_ plasmid, and its sequence was verified by sequencing. Using this vector, the *EZT1* orthologous gene (L203_03018) from *C. depauperatus* CBS7841 was amplified by PCR using primer pair B21896/B21897 and inserted into the vector via Gibson assembly (New England Biolabs, USA). The resulting construct, pHYG-SH-P_*H3*_-Cd*EZT1* plasmid, was linearised using PacI restriction digestion, purified, and introduced into P_*CTR4*_:*EZT1* (YSB9280). Stable transformants were selected on YPD plates containing hygromycin and confirmed with diagnostic PCR. Overexpression of *CdEZT1* was confirmed by qRT-PCR.

## Supplementary Material

Supplementary Files

This is a list of supplementary files associated with this preprint. Click to download.

- SupplementaryData6.xlsx
- SupplementaryData4.xlsx
- SupplementaryData3.xlsx
- SupplementaryData5.xlsx
- SupplementaryData2.xlsx
- SupplementaryInformation.pdf
- SupplementaryData1.xlsx

## Data Availability

Source data and analysis scripts are available on figshare (10.6084/m9.figshare.32099587; reviewer link: https://figshare.com/s/98010b587be78c73cc17). RNA-seq and ChIP-seq data have been deposited in the Gene Expression Omnibus (GEO) under accession numbers GSE328125 and GSE328126, respectively. These datasets are available for peer review using the following tokens: RNA-seq, efeviayejjyjtcz; ChIP-seq, mncheqearjcplqv. WGS data have been deposited in NCBI BioProject under accession number PRJNA1459063 (available for peer review at https://dataview.ncbi.nlm.nih.gov/object/PRJNA1459063?reviewer=v74j1876c6266rg3j1ijvn1bck). All deposited datasets will be publicly available upon publication.
